# Bridging CNNs and vision transformers for efficient tea leaf phytopathogen diagnosis: GL-MobFormer

**DOI:** 10.3389/fpls.2026.1814962

**Published:** 2026-05-12

**Authors:** Yuntong Yang, Xiaosong Li, Qinzi Li, Yong Sun, Chunjing Yang, Hongxu Chen, Xiaoqing Yuan, Can Hu

**Affiliations:** 1 Deyang Agricultural College, De’ yang, China; 2 Tongren Academy of Agricultural Sciences, Tong’ ren, China; 3 Sichuan Agricultural University, Ya’ an, China

**Keywords:** *Camellia sinensis*, edge computing, explainable AI (XAI), lightweight neural networks, phytopathology, precision agriculture

## Abstract

**Background:**

Accurate identification of visible disease symptoms is essential for the sustainable management of tea (*Camellia sinensis*) cultivation. However, balancing high diagnostic accuracy with the computational efficiency required for deployment on agricultural edge devices remains a significant challenge.

**Methods:**

We propose GL-MobFormer, a lightweight hybrid deep learning framework. This architecture integrates the local feature extraction capabilities of MobileNetV3 with the global contextual modeling of a Transformer Encoder. To improve model robustness in unstructured field environments, we applied the CutMix data augmentation strategy. The framework was evaluated on a dataset comprising 5,278 tea leaf images across seven phytosanitary categories.

**Results:**

Empirical evaluations demonstrate that GL-MobFormer achieved a classification accuracy of 95.13% and a Matthews Correlation Coefficient (MCC) of 0.9417. Crucially, this performance was maintained with a low computational footprint of merely 0.33 G FLOPs(Floating Point Operations). Importantly, an occlusion-based sensitivity protocol was implemented to provide quantitative grounding for model interpretability. Results revealed that systematically masking only the top 5% of critical activation regions led to an average reduction of 70.61% in classification confidence, empirically confirming that the model’s diagnostic logic is faithfully anchored on pathologically relevant lesion features rather than background noise.

**Conclusion:**

GL-MobFormer achieves an optimal trade-off between diagnostic precision and computational overhead. It provides a practical and highly efficient solution for on-site, real-time phytosanitary monitoring in precision agriculture.

## 1 Introduction

### 1.1 Economic significance and the threat of phytopathogens

Tea (*Camellia sinensis*) is a globally significant cash crop and constitutes a crucial pillar of the agricultural economy, particularly in developing regions (Ali Abaker Omer et al., 2025; Kumbasaroglu, 2026). However, the cultivation of high-quality tea is continuously threatened by a spectrum of foliar diseases, including Algal Leaf Spot, Brown Blight, and Gray Blight (Datta and Gupta, 2023). These pathological conditions not only damage the photosynthetic apparatus and reduce biomass accumulation, but also disrupt the secondary metabolite profiles of the leaves, ultimately compromising the sensory quality and commercial value of the harvest (Datta and Gupta, 2023; Jan et al., 2021). Therefore, establishing rapid, accurate, and automated disease diagnosis protocols is critical for implementing timely phytosanitary interventions and advancing sustainable precision agriculture (Murugesan et al., 2025).

### 1.2 Limitations of conventional and early deep learning paradigms

Traditionally, the identification of phytopathogens has relied on visual inspection by agricultural experts (Li et al., 2025). This manual paradigm is inherently limited by human subjectivity, high labor costs, and a heightened susceptibility to misdiagnosis, particularly during the early, visually subtle stages of infection (Ferentinos, 2018). It is critical to define the scope of computer vision-based diagnosis in this context: the current framework is primarily engineered to identify visible symptomatic stages rather than latent infections at the physiological or biochemical levels. While the application of Convolutional Neural Networks (CNNs) has significantly advanced automated crop disease (Kamilaris and Prenafeta-Boldú, 2018; Too et al., 2019; Liu and Wang, 2021), inherent structural constraints remain. Standard CNNs excel at extracting localized spatial hierarchies and fine-grained textural features; however, their restricted receptive fields limit their capacity to aggregate long-range dependencies and global contextual information across the entire leaf surface (Wang et al., 2024). This limitation frequently results in classification errors when models encounter different fungal infections that manifest morphologically similar necrotic lesions, or when processing images captured under unstructured field environments with complex backgrounds (Long and Wang, 2025).

### 1.3 The dilemma of vision transformers in resource-constrained scenarios

To overcome the locality bias of CNNs, Vision Transformer (ViT) architectures have been widely adopted (Khan et al., 2023), utilizing self-attention mechanisms to establish global relational modeling (Vaswani et al., 2017). Despite their powerful contextual capabilities, pure ViT models typically require massive datasets for pre-training to converge effectively and involve substantial computational overhead (Touvron et al., 2020; Yuan et al., 2021). This high parametric complexity fundamentally precludes their seamless integration into mobile or embedded edge devices, which are the primary hardware platforms for real-time, on-site disease monitoring in modern tea plantations (Li et al., 2023; Dai et al., 2025; Ouamane et al., 2025). Consequently, there is an urgent requirement for a hybrid framework that simultaneously ensures high diagnostic accuracy and maintains a low computational footprint (Alawfi, 2025).

### 1.4 The proposed GL-mobformer framework and key contributions

**Figure 1:**
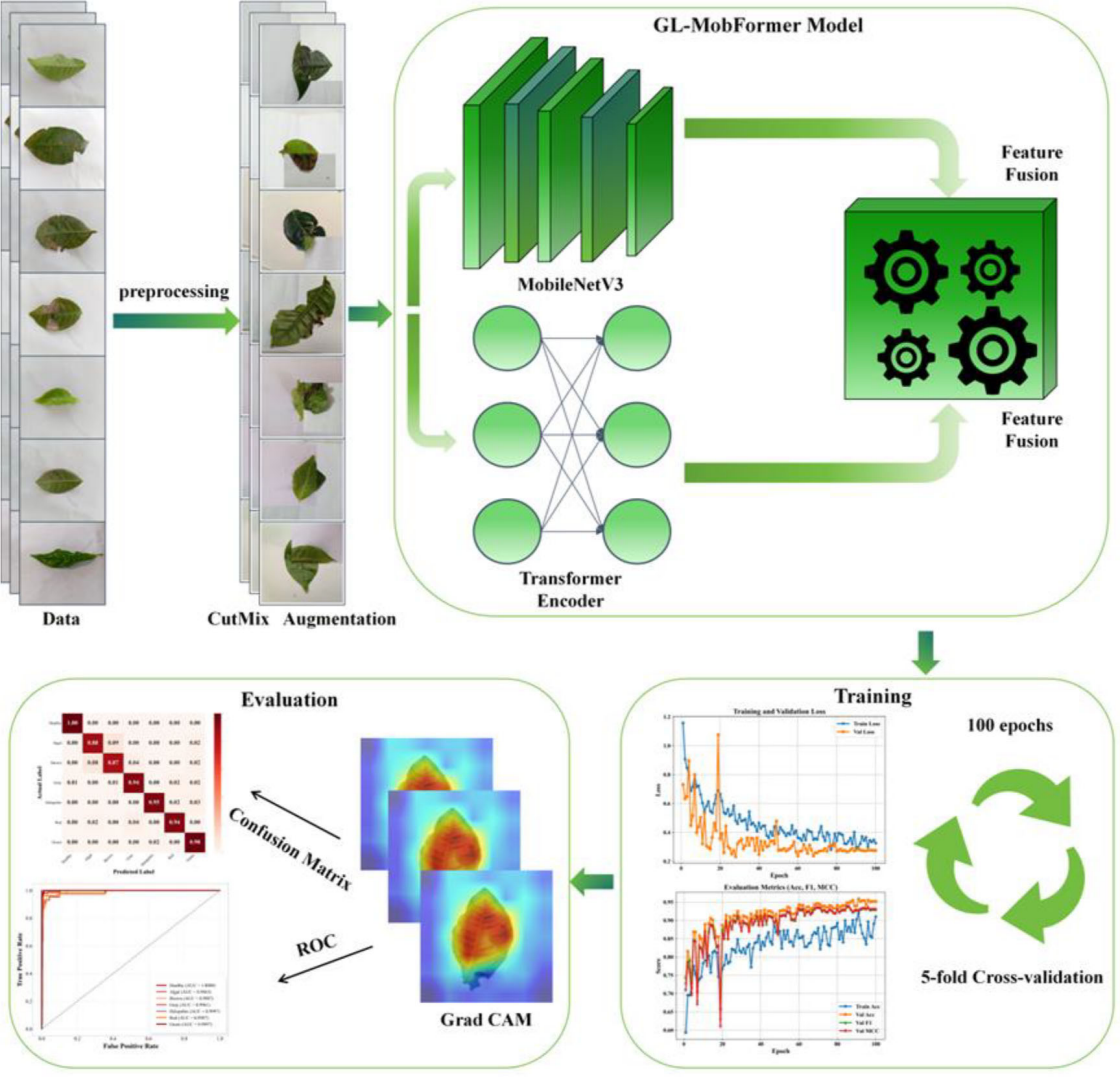
Overall workflow of the GL-MobFormer-based tea leaf disease recognition system.

To bridge these technical gaps, this study introduces GL-MobFormer, a lightweight hybrid framework specifically optimized for tea leaf phytopathogen diagnosis. While recent hybrid CNN-Transformer architectures, such as MobileViT (Mehta and Rastegari, 2021) and FastViT (Vasu et al., 2023), have successfully integrated local and global features for plant pathology, GL-MobFormer distinguishes itself through a parallel dual-branch topology that concurrently processes local textural cues and global spatial dependencies. Unlike sequential hybrids that interleave convolution and attention layers—often leading to increased computational latency on edge devices due to frequent feature map reshaping and memory access—our parallel design explicitly decouples the feature extraction streams. This ensures high diagnostic sensitivity for subtle, low-contrast necrotic margins while maintaining an extremely low operational footprint of 0.33 G FLOPs(Floating Point Operations). By synergizing the inductive bias of MobileNetV3 (Howard et al., 2019) with the global relational modeling of a Transformer Encoder, and further enhancing robustness via the CutMix strategy to prioritize intrinsic pathological markers, the proposed framework achieves a superior trade-off between diagnostic precision and deployment efficiency compared to contemporary hybrid benchmarks. The overall workflow of the proposed diagnostic system is illustrated in **Figure 1**.

The primary contributions of this research are summarized as follows:

1. Architectural Synergy (GL-MobFormer):We developed a dual-branch network topology that concurrently processes local textural cues and global spatial contexts. This integration effectively mitigates the limitations of utilizing homogeneous architectures (pure CNNs or pure ViTs), enhancing the model’s sensitivity across heterogeneous disease morphologies.
2. Enhanced Robustness via CutMix: To bridge the gap between controlled datasets and unstructured environments, the CutMix strategy was incorporated (Yun et al., 2019). This approach encourages the network to prioritize intrinsic pathological markers (e.g., lesion margins) rather than relying on global leaf contours, providing a foundational baseline for future field deployment under variable illumination and occlusions.
3. Efficiency for Edge Deployment: GL-MobFormer achieves an optimal trade-off between predictive accuracy (95.13%) and operational efficiency (0.33 G FLOPs). This extremely low computational demand facilitates its practical deployment on resource-constrained agricultural devices.
4. Biological Interpretability: By employing Grad-CAM (Selvaraju et al., 2016) and LIME (Ribeiro et al., 2016), we quantitatively and visually grounded the model’s decision-making logic. The analysis ensures that classifications are accurately anchored on biologically relevant pathological zones, providing the transparency required for reliable agronomic decision-making.

## 2 Materials and methods

### 2.1 Dataset acquisition and characterization

The dataset employed in this study, teaLeafBD, was sourced from the Mendeley Data repository (Alam et al., 2025). It comprises a comprehensive collection of tea leaf images representing seven distinct phytosanitary categories: Healthy Leaf, Algal Leaf Spot, Brown Blight, Gray Blight, Helopeltis (Tea Mosquito Bug), Red Spider Mite, and Green Mirid Bug. To ensure robust model evaluation and prevent data leakage, the dataset was randomly partitioned into training, validation, and hold-out testing subsets with a distribution ratio of approximately 7:2:1. The specific taxonomic distribution and sample sizes for each category are detailed in **Table 1**.

**Table 1:** Distribution of the tea leaf disease dataset across training, validation, and testing sets.

Category	Train	Val	Test	Total
Healthy leaf	654	187	94	935
Tea algal leaf spot	292	83	43	418
Brown Blight	355	101	52	508
Gray Blight	709	202	102	1013
Helopeltis	424	121	62	607
Red spider	360	103	52	515
Green mirid bug	897	256	129	1282

### 2.2 Experimental setup and training strategy

The GL-MobFormer framework was implemented using the PyTorch 2.0 library (Paszke et al., 2019). Network optimization was conducted via the AdamW optimizer (Loshchilov and Hutter, 2017) with a weight decay of $1\times 10^{-4}$. The initial learning rate was set to 0.001 and modulated by a cosine annealing scheduler over 100 epochs. All experiments were executed on an NVIDIA RTX 4090 GPU to ensure consistent computational conditions across the 5-fold cross-validation process.

### 2.3 Overall diagnostic pipeline and CutMix augmentation

The operational workflow of the proposed intelligent diagnostic framework encompasses four primary phases: image preprocessing, hybrid architectural feature extraction, cross-validation training, and multi-dimensional performance evaluation (illustrated in **Figure 1**).

To mitigate the risk of overfitting—a common constraint for deep networks operating in complex, unstructured agricultural environments—the CutMix data augmentation strategy was implemented during the preprocessing phase.

Given two training samples $\left({\text{x}}_{\text{A}},{\text{y}}_{\text{A}})\text{and}({\text{x}}_{\text{B}},{\text{y}}_{\text{B}}\right)$, the composite sample $\left(\tilde{\text{x}},\tilde{\text{y}}\right)$ is generated as:


$$\tilde{\text{x}}=\text{M}⊙{\text{x}}_{\text{A}}+(1-\text{M})⊙{\text{x}}_{\text{B}}$$

$$\tilde{\text{y}}={\text{λy}}_{\text{A}}+(1-\text{λ}){\text{y}}_{\text{B}}$$

where $\text{M}\in {\left\{0,1\right\}}^{\text{W}\times \text{H}}$ is a binary mask indicating where to drop out and fill in from two images, and λ is the proportion of the combined regions, sampled from a Beta distribution. This technique forces the model to identify localized necrotic margins rather than relying on global leaf contours.

**Figure 2:**
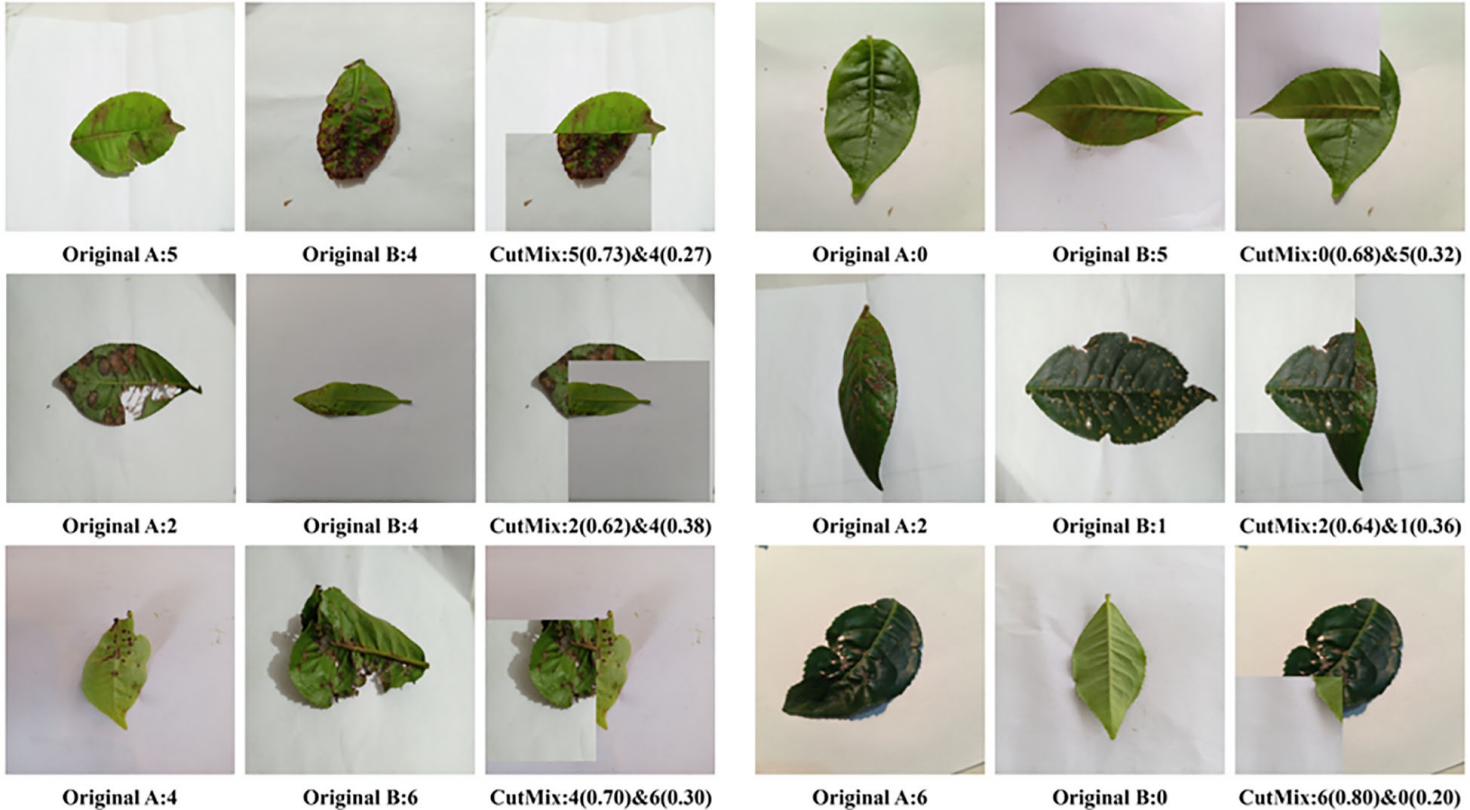
Overview of original tea leaf images and composite samples generated via the CutMix data augmentation strategy.

Unlike traditional geometric transformations, CutMix operates by excising a rectangular spatial region from one training image and pasting it onto another, concurrently blending their ground-truth labels proportionally to the area of the excised patches (Pan et al., 2024). As illustrated in **Figure 2**, this strategy generates diverse composite samples, such as chimeras combining healthy tissues with pest-induced damage, or inter-class blends between morphologically similar fungal infections (e.g., Brown Blight and Gray Blight). By disrupting the continuity of global leaf structures and presenting the network with localized, co-occurring pathological features, this mechanism compels the model to decouple lesions from their original environmental backgrounds (Li et al., 2021). Consequently, the network is forced to prioritize intrinsic diagnostic motifs—such as necrotic margins and specific fungal textures—thereby substantially enhancing its robustness against partial occlusions and complex, overlapping symptoms in unstructured field environments.

### 2.4 The proposed GL-mobformer architecture

To bridge the gap between high diagnostic sensitivity and computational parsimony required for edge deployment, this study introduces GL-MobFormer, a hybrid topology that synergizes the inductive bias of Convolutional Neural Networks (CNNs) with the global relational modeling of Vision Transformers (ViTs).

**Figure 3:**
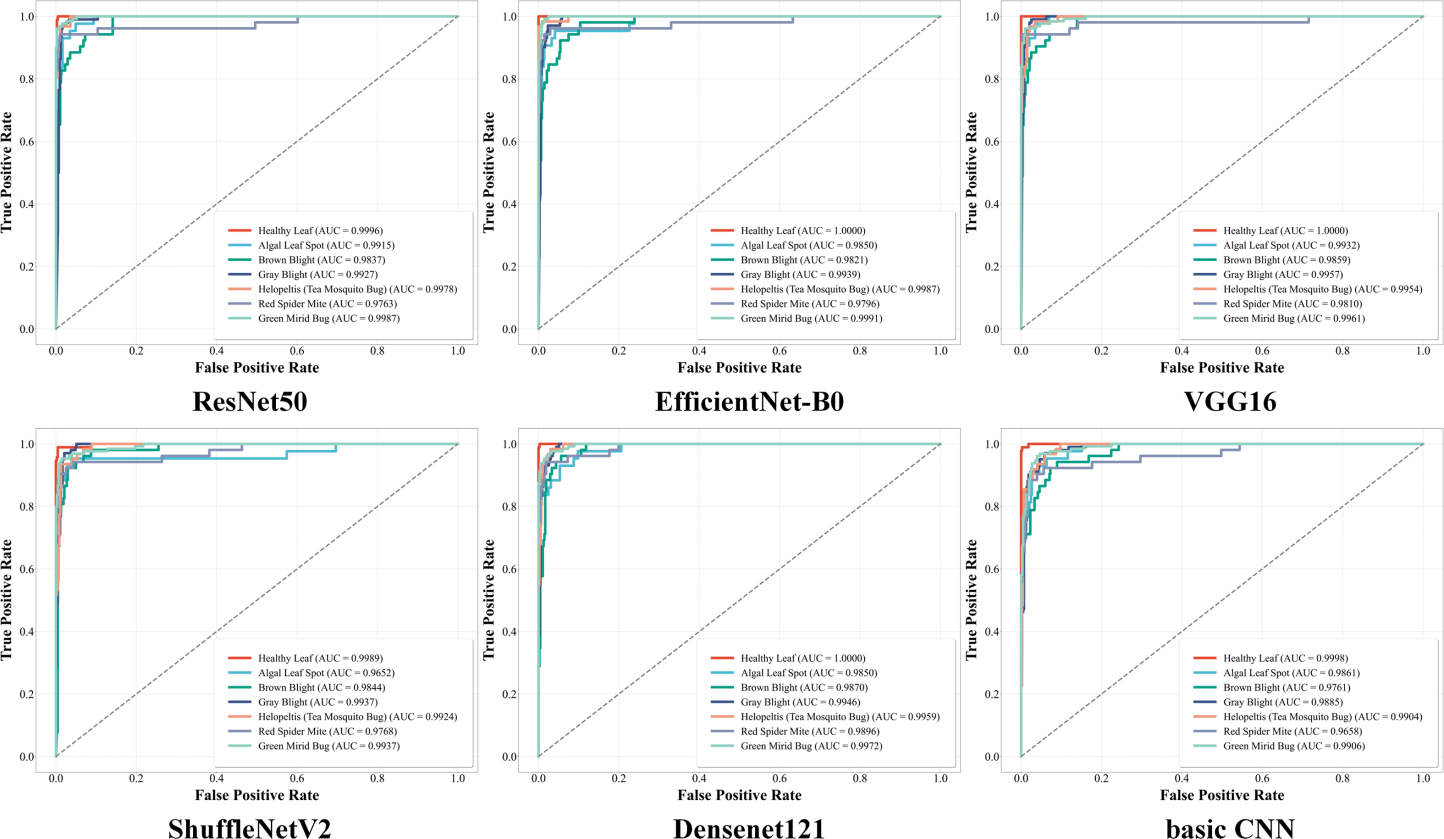
Schematic diagram of the GL-MobFormer model architecture.

As depicted in the data flow of **Figure 3**, the architecture receives 224 × 224 × 3 input images, utilizing a lightweight MobileNetV3 backbone to extract preliminary feature maps with dimensions of 7 × 7 × 960. The system subsequently employs a dual-branch strategy for feature disentanglement:

1. Local Branch: Utilizes Adaptive Average Pooling to aggregate fine-grained textural motifs into a condensed 960-dimensional local feature vector.
2. Global Branch: Employs a Transformer EncoderLite module configured with a single encoder layer L=1 and an embedding dimension of $960\;({\text{d}}_{\text{model}}=960)$. The core of this module is the Multi-Head Self-Attention (MHSA) mechanism utilizing 4 attention heads h=4, which captures long-range spatial dependencies across the leaf surface. The attention mechanism is defined as:

$$\text{Attention}(\text{Q},\text{K},\text{V})=\text{softmax}(\frac{{\text{QK}}^{\text{T}}}{\sqrt{{\text{d}}_{\text{k}}}})\text{V}$$

where Q, K, V represent the Query, Key, and Value matrices, and ${\text{d}}_{\text{k}}$ denotes the scaling factor based on the head dimension. The position-wise feed-forward network (FFN) within this module maintains a hidden dimension of 960 with GELU activation to preserve computational efficiency. Finally, the features from both branches are concatenated into a 1920-dimensional joint representation and mapped to seven phytosanitary categories through a linear classification layer.

### 2.5 Performance metrics

To comprehensively evaluate the diagnostic efficacy of GL-MobFormer, we employed multiple statistical metrics including Accuracy (ACC), F1-score (Sokolova and Lapalme, 2009), and the Matthews Correlation Coefficient (MCC) (Chicco and Jurman, 2020). These metrics are calculated based on the confusion matrix components: True Positives (TP), True Negatives (TN), False Positives (FP), and False Negatives (FN).

Accuracy, reflecting the overall proportion of correctly identified samples, is defined as:


$$\text{ACC}=\frac{\text{TP}+\text{TN}}{\text{TP}+\text{TN}+\text{FP}+\text{FN}}$$

The F1-score provides a harmonic mean of Precision and Recall, ensuring a balanced assessment in the presence of imbalanced class distributions:


$$\text{F}1=2\times \frac{\text{Precision}\times \text{Recall}}{\text{Precision}+\text{Recall}}$$

where $\text{Precision}=\frac{\text{TP}}{\text{TP}+\text{FP}}$ and $\text{Recall}=\frac{\text{TP}}{\text{TP}+\text{FN}}$.

To provide a rigorous evaluation of the model’s performance across seven phytosanitary categories, the Matthews Correl (Ayon et al., 2024). For the multiclass classification task in this study, MCC is calculated using the following equation:


$$\text{MCC}=\frac{\text{c}\times \text{s}-\sum_{\text{k}=1}^{\text{K}}{\text{p}}_{\text{k}}{\text{t}}_{\text{k}}}{\sqrt{\left({\text{s}}^{2}-\sum_{\text{k}=1}^{\text{K}}{\text{p}}_{\text{k}}^{2})({\text{s}}^{2}-\sum_{\text{k}=1}^{\text{K}}{\text{t}}_{\text{k}}^{2}\right)}}$$

where s denotes the total number of samples, c represents the total number of correctly predicted samples, and ${\text{t}}_{\text{k}}$ and ${\text{p}}_{\text{k}}$ correspond to the ground-truth and predicted occurrences of class k, respectively. This metric ranges from -1 to 1, where 1 indicates a perfect prediction and 0 reflects a performance no better than random guessing.

Finally, the network optimization was guided by the categorical cross-entropy loss function (LCE):


$$L\text{CE}=-\overset{\text{C}}{\underset{\text{i}=1}{\sum }}{\text{y}}_{\text{i}}\log({\hat{\text{y}}}_{\text{i}})$$

where ${\text{y}}_{\text{i}}$ represents the one-hot encoded ground truth and ${\hat{\text{y}}}_{\text{i}}$ is the predicted probability for category i.

### 2.6 Model interpretability and faithfulness analysis

Recognizing the imperative for transparency in automated agricultural diagnostics, we implemented a multi-faceted interpretability framework integrating Gradient-weighted Class Activation Mapping (Grad-CAM), Local Interpretable Model-agnostic Explanations (LIME), and quantitative occlusion sensitivity analysis. Grad-CAM leverages the gradient flow into the final convolutional layer to generate spatial heatmaps, identifying regions critical to the model’s diagnostic logic. Concurrently, LIME provides superpixel-based segmentation to isolate specific pathological motifs, such as fungal lesions or arthropod-induced damage. To bridge the gap between qualitative visualization and empirical evidence, we further conducted occlusion-based sensitivity tests to quantitatively assess the faithfulness of these representations. This comprehensive approach ensures that GL-MobFormer’s predictions are rigorously grounded in biologically relevant morphological characteristics, thereby mitigating the risk of decision-making based on dataset biases or background artifacts.

## 3 Results

### 3.1 Training dynamics and cross-validation evaluation

**Figure 4:**
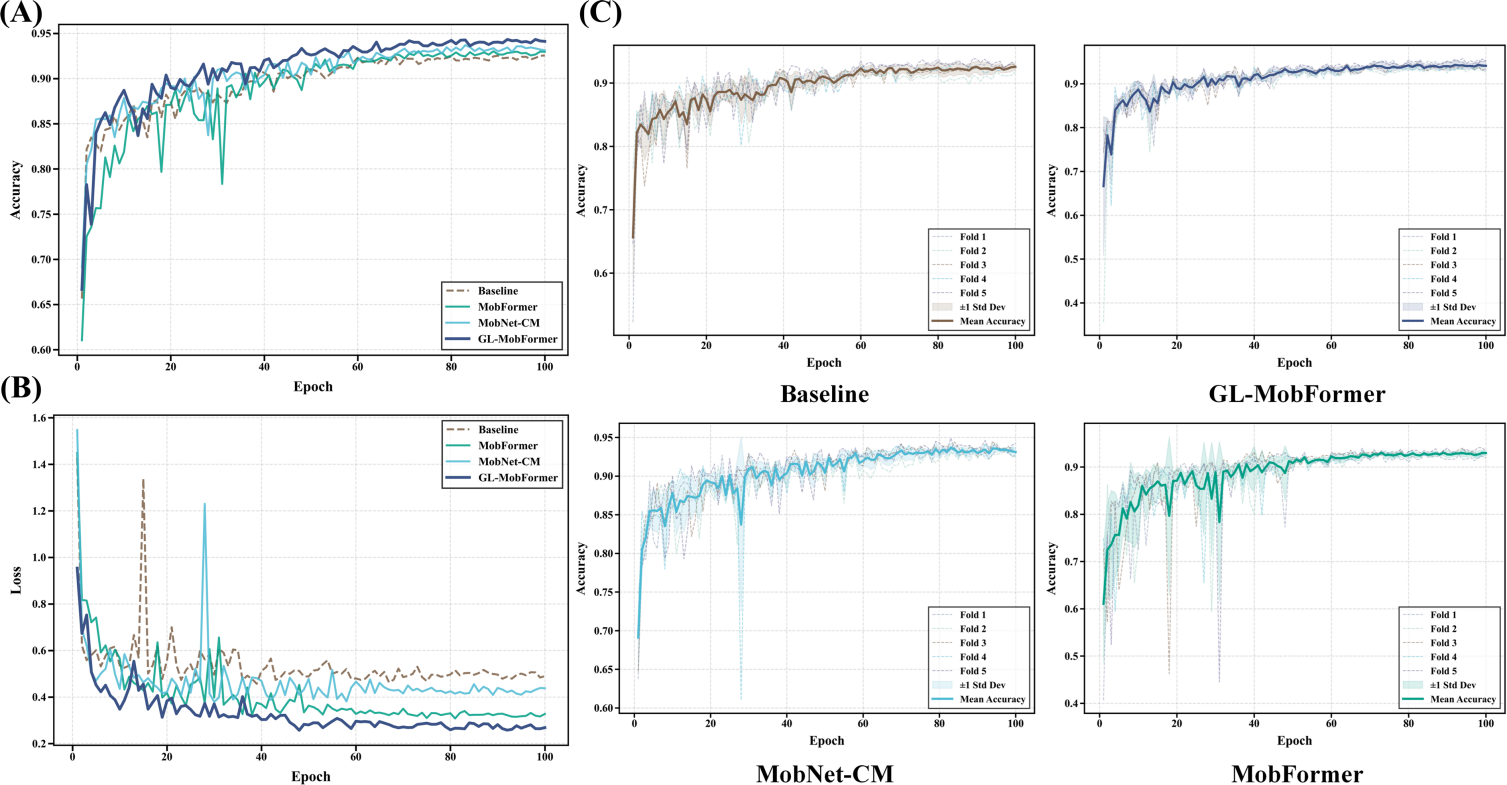
Comparative training dynamics of GL-MobFormer and ablation variants under 5-fold cross-validation. **(A)** Mean validation accuracy; **(B)** Mean validation loss; **(C)** Fold-wise trajectories for Baseline, GL-MobFormer, MobNet-CM, and MobFormer. Shaded regions and dashed lines denote standard deviation and individual fold performance, respectively.

To rigorously validate the statistical reliability of the proposed framework and mitigate any potential evaluation bias arising from random data partitioning, the training process was executed utilizing a 5-fold cross-validation paradigm. Analysis of the optimization trajectories reveals that the GL-MobFormer architecture possesses a stable convergence profile and consistent gradient updates. As illustrated by the validation loss curves in **Figures 4B**, GL-MobFormer continuously maintained a lower categorical cross-entropy loss compared to both the baseline and intermediate ablation architectures. Notably, the proposed model exhibited a rapid, steady descent in the loss function during the initial 30 epochs, followed by a steady-state convergence with minimal oscillations. This trajectory indicates an effective penalization of misclassifications, facilitated by the multi-scale feature space generated by the hybrid CNN-Transformer design.

Furthermore, as corroborated by **Figure 4A** and the detailed fold-wise dynamics presented in **Figure 4C**, the model attained a peak mean validation accuracy of 94.79% ± 0.74%. The notably narrow standard deviation band across all five distinct folds provides quantitative validation of the hybrid architecture’s enhanced robustness. It confirms a reduced sensitivity to variations in image distribution within complex, unstructured agricultural datasets. This stability underscores that the integration of global attention mechanisms effectively mitigates the risk of overfitting, ensuring consistent diagnostic performance across heterogeneous leaf samples.

### 3.2 Ablation study on architectural components

An extensive ablation study was conducted to quantitatively isolate and evaluate the discrete contributions of the lightweight CNN backbone, the Transformer global spatial module, and the CutMix data augmentation strategy. As summarized in **Table 2**, an incremental improvement in predictive capabilities was observed upon the sequential integration of these components. The Baseline model (pure MobileNetV3 configuration) yielded an accuracy of 0.9304. The independent integration of the Transformer module (MobFormer) and the CutMix augmentation (MobNet-CM) enhanced the accuracy to 0.9349 and 0.9416, respectively.

**Table 2:** Performance comparison of ablation models using 5-fold cross-validation (Mean ± Standard Deviation).

Model	Accuracy	F1-score	MCC	Loss
Baseline	0.9304 ± 0.0086	0.9158 ± 0.0113	0.9167 ± 0.0102	0.4751 ± 0.0911
MobFormer	0.9349 ± 0.0060	0.9214 ± 0.0063	0.9220 ± 0.0072	0.3081 ± 0.0483
MobNet-CM	0.9416 ± 0.0057	0.9301 ± 0.0062	0.9302 ± 0.0069	0.3858 ± 0.0662
GL-MobFormer	0.9485 ± 0.0042**	0.9361 ± 0.0055*	0.9383 ± 0.0061**	0.2583 ± 0.0464**

** denotes p < 0.01; * denotes p < 0.05 compared to Baseline.

**Figure 5:**
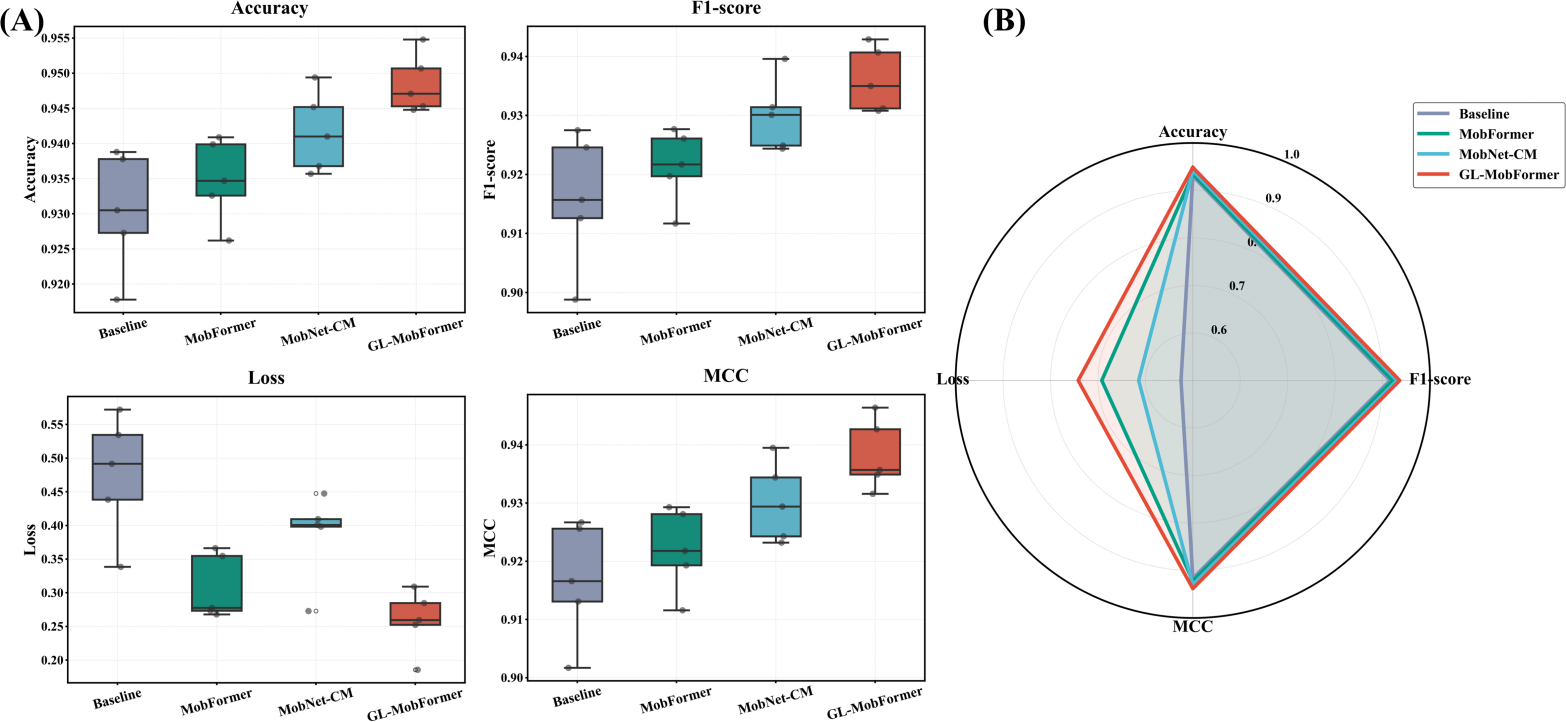
Visualization of performance metrics for ablation models. **(A)** Box plots showing the distribution of Accuracy, F1-score, Loss, and MCC across 5-fold cross-validation. **(B)** Radar chart for comprehensive performance comparison of Baseline, MobFormer, MobNet-CM, and GL-MobFormer.

Crucially, the complete GL-MobFormer framework optimized overall performance, attaining a peak accuracy of 0.9485 and reducing the training loss to 0.2583. Two-tailed T-test analyses confirmed that these improvements across multiple evaluation metrics were statistically significant (p < 0.01) when compared to the baseline configuration. The statistical distribution of these metrics across the 5-fold cross-validation is visually depicted in the box plots of **Figure 5A**. GL-MobFormer consistently registered the highest median values for Accuracy, F1-score, and MCC, while simultaneously maintaining the tightest interquartile range for loss, which highlights its superior predictive stability. Furthermore, the radar chart in **Figure 5B** provides a holistic visual comparison, illustrating that GL-MobFormer forms the outermost performance envelope across all five key dimensions. This comprehensive evaluation verifies that combining global attention mechanisms with localized data augmentation robustly amplifies the model’s discriminative capacity when identifying complex foliar pathologies.

### 3.3 Benchmarking on the independent test set

#### 3.3.1 Performance comparison with ablation variants

**Figure 6:**
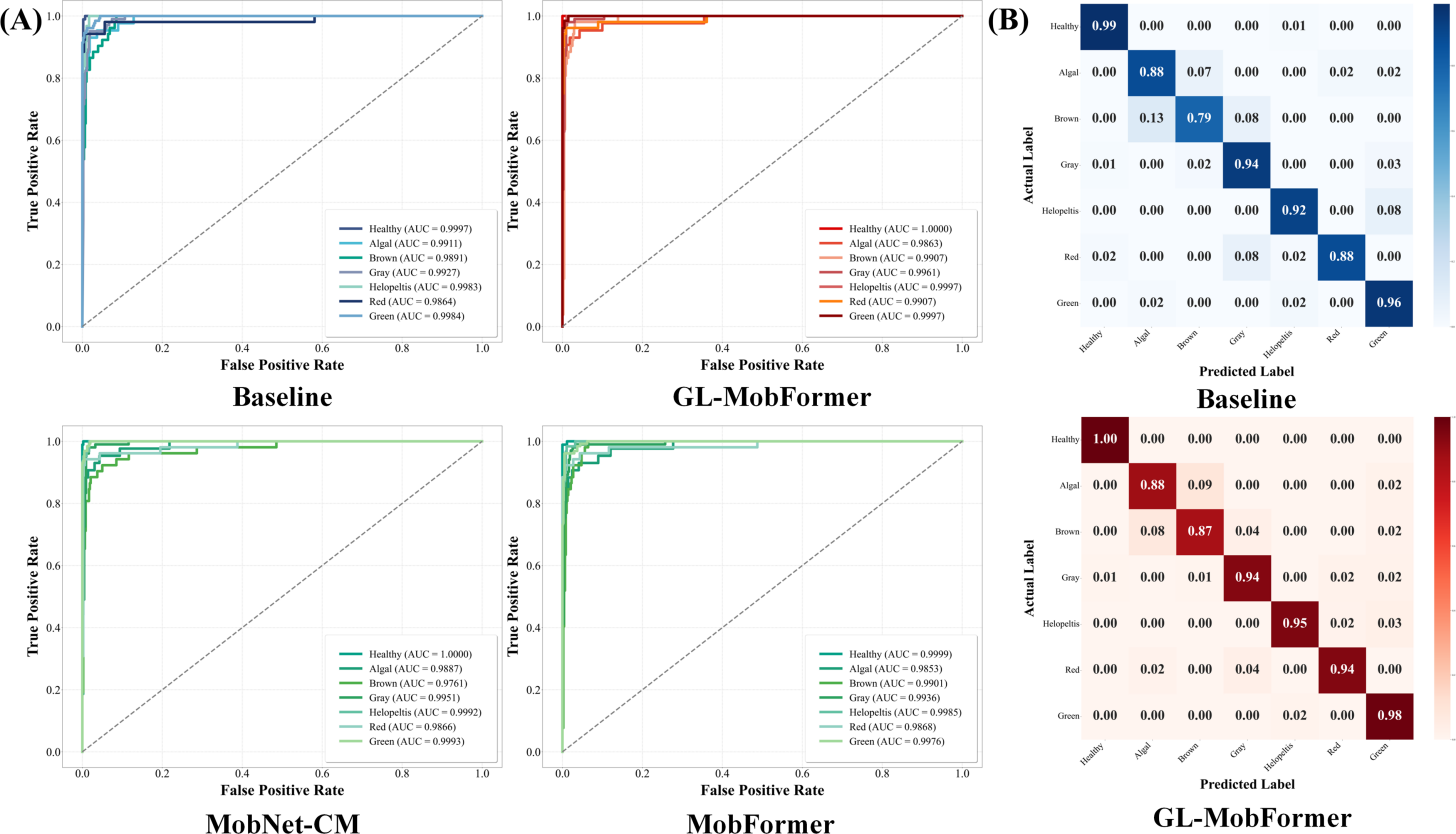
Performance evaluation on the independent test set. **(A)** ROC, Receiver Operating Characteristic curves and corresponding AUC, Area Under the Curve values for the Baseline, MobNet-CM, MobFormer, and the proposed GL-MobFormer across seven categories. **(B)** Confusion matrices for the Baseline model (top) and GL-MobFormer (bottom), illustrating the classification accuracy for each specific category: Healthy Leaf, Algal Leaf Spot, Brown Blight, Gray Blight, Helopeltis, Red Spider Mite, and Green Mirid Bug.

To evaluate the practical generalization capabilities of the models under conditions approximating real-world diagnostic scenarios, assessments were performed using an unseen hold-out test set. As detailed in **Table 3**, GL-MobFormer achieved optimal performance across all evaluated metrics, registering a classification accuracy of 0.9513 and a Matthews Correlation Coefficient (MCC) of 0.9417. Comparative analysis of the Receiver Operating Characteristic (ROC) curves in **Figure 6A** further substantiates the model’s robust discriminative power. GL-MobFormer maintains the highest Area Under the Curve (AUC) for each phytosanitary category, with values approaching 1.00 for Healthy Leaf and Green Mirid Bug damage. This indicates an exceptionally high true positive rate alongside a negligible false positive rate across diverse pathological signatures.Notably, despite the inherent class imbalance within the teaLeafBD dataset—where the Green Mirid Bug category contains significantly more samples than Algal Leaf Spot—the model exhibited balanced diagnostic performance across all categories. The choice of macro-averaged metrics, such as MCC and Macro-F1, ensures that this high performance is not skewed by the majority class, thereby validating the framework’s reliability for minority disease detection.

**Table 3:** Performance comparison of the proposed GL-MobFormer and its ablation variants on the independent test set.

Model	Backbone	Global module	Data aug	Accuracy	Precision (macro)	Recall (macro)	F1-score (macro)	MCC
GL-MobFormer	MobileNetV3	Transformer	CutMix	0.9513	0.9425	0.9384	0.9403	0.9417
MobNet-CM	MobileNetV3	Х	CutMix	0.9419	0.9295	0.9278	0.9286	0.9305
MobFormer	MobileNetV3	Transformer	Standard	0.9307	0.9188	0.9134	0.9150	0.9172
Baseline	MobileNetV3	Х	Standard	0.9270	0.9189	0.9097	0.9132	0.9126

The ablation variants exhibited a marked performance degradation in the absence of the synergistic architectural components. The exclusion of the Transformer module decreased accuracy to 0.9419, and the omission of CutMix reduced it further to 0.9307; the Baseline model yielded the lowest accuracy at 0.9270. These results strongly suggest that while local textural features are foundational, they remain insufficient for high-precision diagnosis in variable field environments. Crucially, the confusion matrices presented in **Figure 6B** demonstrate that GL-MobFormer effectively mitigates misclassifications between diseases that exhibit morphological similarities. Specifically, a slight confusion persists between Brown Blight and Algal Leaf Spot, as both pathogens manifest as dark brown necrotic lesions in their early stages. Biologically, Brown Blight (Colletotrichum gloeosporioides) is characterized by prominent concentric ring patterns and relatively distinct boundaries, whereas Algal Leaf Spot (Cephaleuros virescens) presents a more velvety surface with irregular, feathery margins. The proposed GL-MobFormer successfully captures these subtle differences by leveraging its global contextual modeling to distinguish the rhythmic ‘ring’ structures of Brown Blight from the diffuse, ‘velvety’ texture of Algal Leaf Spot. A detailed analysis of the minority class performance reveals that both the Baseline model and the proposed GL-MobFormer achieved a consistent recall rate of 0.88 for Algal Leaf Spot. This parity suggests that the diagnostic bottleneck for this category stems from localized visual ambiguities rather than a systematic bias induced by sample scarcity. However, while the Baseline model struggled to isolate “Brown Blight” characteristics—misidentifying 13% of samples and yielding an accuracy of merely 0.79—the proposed GL-MobFormer successfully leveraged global contextual information to improve “Brown Blight” recognition accuracy to 0.87. This balanced sensitivity across both majority and minority classes, even without explicit class-weighted loss functions, underscores the inherent robustness of the parallel CNN-Transformer architecture.

#### 3.3.2 Comparative evaluation against state-of-the-art architectures

**Figure 7:**
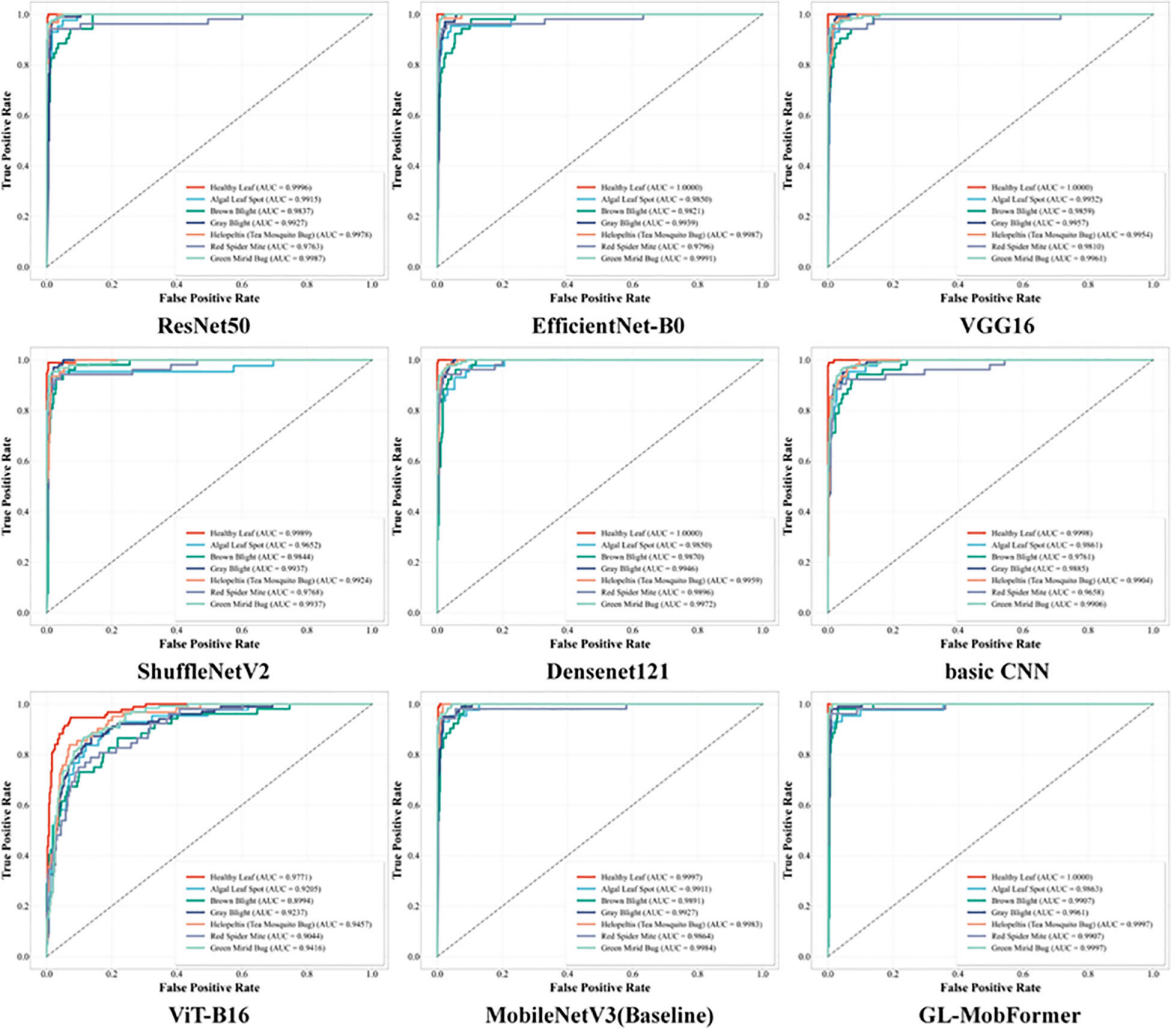
ROC, Receiver Operating Characteristic curves and corresponding AUC, Area Under the Curve values for 9 different deep learning models on the tea leaf disease test set. Each colored curve represents the classification performance of a specific model. Curves closer to the top-left corner with AUC values approaching 1.0 indicate superior comprehensive ability in distinguishing between healthy leaves and various diseases.

The diagnostic precision and operational efficiency of the proposed GL-MobFormer were subsequently benchmarked against a diverse spectrum of mainstream deep learning architectures. Analysis of the ROC curves in **Figure 7** elucidates the trade-off between the True Positive Rate and False Positive Rate across nine competitive baseline models. While all evaluated architectures maintained relatively high AUC values, the trajectory for GL-MobFormer defines the outermost boundary of superiority, adhering closely to the upper-left coordinate of the ROC space. This positioning indicates maximal sensitivity coupled with minimal false alarm rates, yielding a macro-average AUC that approaches 1.0, ranking highest among all evaluated frameworks. This finding highlights the hybrid model’s capacity to maintain rigorous precision regardless of decision threshold variations across different plant disease categories.

As summarized in **Table 4**, GL-MobFormer achieved the highest diagnostic metrics for tea leaf classification within this study, registering a peak accuracy of 95.13% and a macro-averaged F1-score of 0.9403. This represents a substantial improvement over established heavyweight CNN paradigms, including ResNet50 (He et al., 2015) (93.82%), VGG16 (Simonyan and Zisserman, 2014) (93.63%), and the standalone MobileNetV3 (Howard et al., 2019) (92.70%). Furthermore, the proposed model significantly outperformed the pure Vision Transformer ViT-B16 71.16%. This notable performance disparity underscores the efficacy of integrating CNN-based inductive biases; pure self-attention mechanisms lack the inherent capacity to accurately extract localized pathological features when massive pre-training datasets are unavailable, leading to underfitting on specialized botanical datasets.

**Table 4:** Performance comparison of different deep learning models on the dataset.

Model	Accuracy	Precision (macro)	Recall (macro)	F1-score (macro)	MCC	Params (M)	FLOPs (G)
GL-MobFormer	0.9513	0.9425	0.9384	0.9403	0.9417	10.53	0.33
ResNet50	0.9382	0.9234	0.9254	0.9241	0.9261	23.52	4.13
EfficientNet-B0	0.9363	0.9232	0.9192	0.9205	0.9238	4.02	0.41
VGG16	0.9363	0.9224	0.9208	0.9211	0.9239	134.30	15.52
MobileNet-V3	0.9270	0.9189	0.9097	0.9132	0.9126	4.21	0.23
ShuffleNetV2	0.9232	0.9119	0.9074	0.9094	0.9081	1.26	0.15
DenseNet121	0.9082	0.8919	0.8830	0.8864	0.8901	6.96	2.90
Basic CNN	0.8876	0.8783	0.8585	0.8670	0.8654	26.08	0.76
ViT-B16	0.7116	0.6862	0.6658	0.6739	0.6536	85.80	11.29

**Figure 8:**
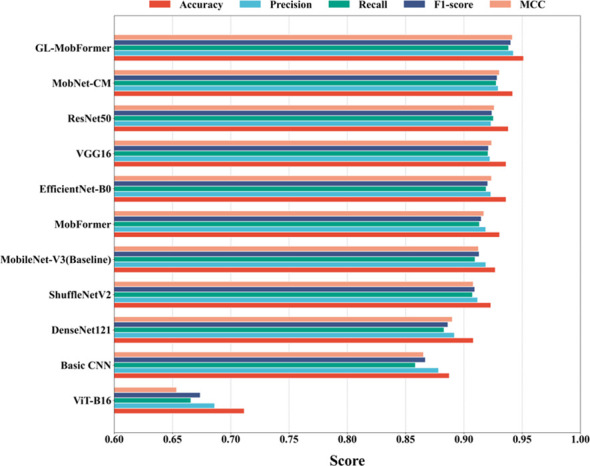
Comprehensive performance comparison of different models on the test set. The bar chart illustrates the performance of 11 different network architectures across five key evaluation metrics: Accuracy, Precision, Recall, F1-score, and MCC, Matthews Correlation Coefficient.

In terms of computational efficiency and hardware deployability, GL-MobFormer achieved a highly favorable balance between predictive sensitivity and model compactness. With a computational footprint of merely 0.33 G FLOPs and 10.53 M parameters, the architecture is significantly more efficient than DenseNet121 (Huang et al., 2016) (2.90 G FLOPs) and ResNet50 (4.13 G FLOPs). Furthermore, even highly optimized scalable networks like EfficientNet-B0 (Tan and Le, 2019) required a slightly higher computational cost (0.41 G FLOPs) while yielding a lower accuracy (93.63%). While ShuffleNetV2 (Ma et al., 2018) remains the most computationally sparse network evaluated (0.15 G FLOPs), it incurred a 2.81% accuracy deficit compared to GL-MobFormer. A comprehensive horizontal evaluation across 11 distinct models, detailed in **Figure 8** and **Table 4**, confirms these findings. GL-MobFormer consistently ranks highest across all five primary evaluation dimensions. These results demonstrate that the framework successfully addresses the typical structural trade-off between lightweight operational efficiency and high-fidelity diagnostic precision, making it highly suitable for integration into resource-constrained agricultural edge devices.

### 3.4 Biological interpretability of the decision logic

To ensure the diagnostic reliability and operational transparency of GL-MobFormer for practical agronomic deployment, a dual-layer qualitative interpretability analysis was performed utilizing Gradient-weighted Class Activation Mapping (Grad-CAM) and Local Interpretable Model-agnostic Explanations (LIME). Given the stringent requirements for accountability in precision agriculture, visualizing the model’s decision-making logic is essential to confirm that classifications are derived from genuine pathological evidence rather than dataset artifacts or background biases.

**Figure 9:**
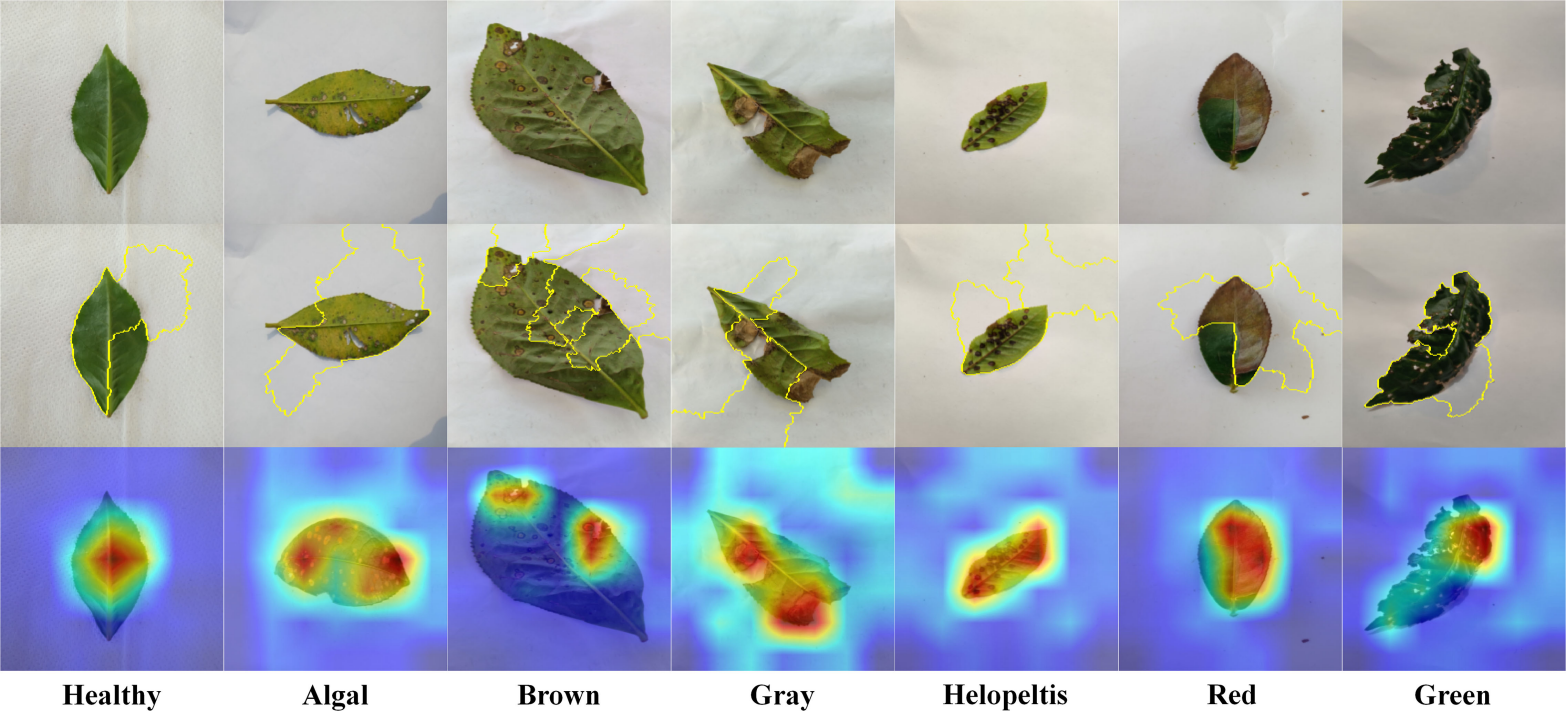
Interpretability visualization analysis of the GL-MobFormer model based on Grad-CAM and LIME across seven phytosanitary categories.

As illustrated in **Figure 9**, visualizations across seven distinct categories—ranging from fungal infections (e.g., Brown Blight) to arthropod-induced mechanical damage (e.g., Red Spider Mite and Green Mirid Bug)—demonstrate that the network accurately targets relevant necrotic zones and foliar defects. Specifically, the Grad-CAM heatmaps consistently display high activation peaks (red focal zones) precisely centered on symptomatic lesions. Simultaneously, the LIME superpixel segmentations (indicated by yellow boundaries) successfully isolate the specific textural motifs that contribute most heavily to the positive classification, systematically filtering out non-informative healthy tissue and background pixels. Crucially, a pronounced spatial convergence is observed between the high-contrast activation peaks generated by Grad-CAM and the localized superpixel boundaries mapped by LIME. This alignment unequivocally verifies that the classification logic is anchored in biologically meaningful morphological characteristics, such as the characteristic chlorotic halos and necrotic centers typical of specific fungal pathogens. Such spatial interpretability confirms that GL-MobFormer is robust against environmental noise and visual interference, validating the scientific rigor of the hybrid architecture for real-time foliar disease monitoring.

**Figure 10:**
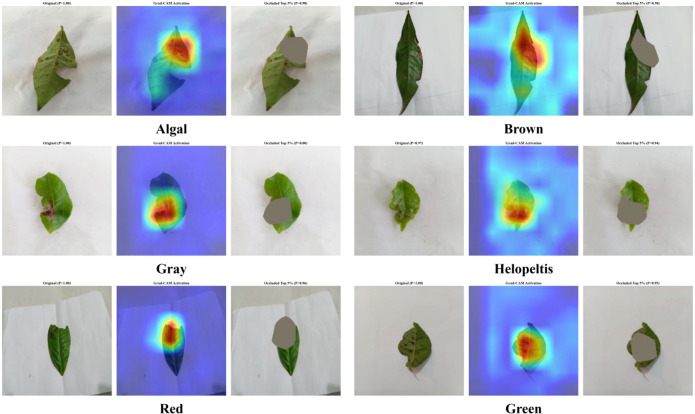
Qualitative interpretability maps for tea leaf diseases. The three-panel visualization for each category displays the original image, the Grad-CAM activation heatmap, and the 5% top-activation occlusion mask used for quantitative validation.

To provide a quantitative basis for the qualitative visualizations, we performed an occlusion sensitivity analysis to evaluate the “faithfulness” of the model’s internal representations (Zeiler and Fergus, 2013). This procedure systematically masked the top 5% of pixels characterized by the highest Grad-CAM activation values, subsequently measuring the resultant attenuation in classification confidence. As illustrated by the three-panel diagnostic maps in **Figure 10** (displaying the original image, activation heatmap, and 5% occlusion mask) and the statistical metrics in **Table 5**, the occlusion of these critical regions led to a significant reduction in diagnostic confidence across the test set, with a mean decrease of 70.61%.

**Table 5:** Statistical summary of prediction confidence reduction post-occlusion.

Category	Confidence drop	Status
Healthy	60.68	Moderate Sensitivity
Algal	68.42	Moderate Sensitivity
Brown	54.46	Moderate Sensitivity
Gray	83.92	High Sensitivity
Helopeltis	52.50	Moderate Sensitivity
Red	70.52	Moderate Sensitivity
Green	82.70	High Sensitivity
Overall Average	70.61	–

Notably, the Gray Leaf Spot and Green Algal Spot categories exhibited the most pronounced sensitivity, with confidence scores declining by 83.92% and 82.70%, respectively. Even for the Healthy class, a confidence reduction of 60.68% was observed, suggesting that the model verifies the absence of pathogens by assessing the global structural integrity and textural homogeneity of the foliar surface. These empirical findings, consistent with the principles of local interpretable explanations, demonstrate that GL-MobFormer effectively prioritizes pathologically relevant features. This localized focus ensures robust performance amidst the complex environmental noise and visual interference inherent in real-time monitoring scenarios.

## 4 Discussion

### 4.1 Overview of principal findings

The transition from manual visual inspection to automated, objective phytopathogen diagnosis is a foundational step for implementing timely interventions in precision agriculture. This study developed and evaluated GL-MobFormer, a hybrid neural network architecture designed to address the specific computational and diagnostic challenges encountered in unstructured field environments. Through rigorous empirical evaluation on a hold-out test set, the proposed model demonstrated a superior classification accuracy of 95.13%. Beyond standard performance metrics, the integration of dual-layer interpretability analyses (Grad-CAM and LIME) provided essential validation that the network’s feature extraction process is correctly anchored on biologically relevant morphological anomalies, such as necrotic lesions and pest-induced structural damage, rather than being biased by background artifacts. Crucially, the results from the occlusion sensitivity analysis provide empirical grounding for these visual interpretations. The observed 70.61% average reduction in prediction confidence upon masking only 5% of critical diagnostic regions quantitatively demonstrates the high “faithfulness” of the model. This sensitivity suggests that GL-MobFormer has effectively internalized the discriminative textural and spatial signatures of various tea diseases, rather than relying on global image statistics. Such robust and verifiable interpretability is paramount for the operational deployment of deep learning models in agriculture, where diagnostic accountability and resistance to environmental noise are as critical as raw accuracy.

### 4.2 The inevitability of fusing local and global representations

A persistent challenge in automated plant phenotyping is the reliable differentiation of fungal infections that produce morphologically similar visual symptoms. Traditional Convolutional Neural Networks (CNNs) demonstrate strong inductive biases (Picón et al., 2019), making them highly effective at capturing fine-grained localized spatial hierarchies and textural motifs, such as the concentric rings or specific margin patterns of a fungal lesion. However, their restricted receptive fields inherently limit their capacity to aggregate long-range dependencies and holistic contextual information across the entire anatomical structure of the leaf. Conversely, while pure Vision Transformers excel at global relational modeling (Guan et al., 2025) through self-attention mechanisms, their reliance on massive pre-training datasets often leads to severe overfitting (Yao et al., 2022) and suboptimal feature extraction when applied to specialized, moderately sized botanical datasets.

Unlike contemporary hybrid architectures such as MobileViT, which typically employ a sequential or interleaved design by alternating convolution and attention layers, GL-MobFormer utilizes a parallel dual-branch topology. In sequential hybrids, feature maps undergo frequent transformations between spatial and latent representations. This process can lead to a cumulative loss of the fine-grained textural information required for identifying subtle necrotic margins in tea leaves. Furthermore, the frequent tensor reshaping and memory access operations inherent to sequential designs often incur computational bottlenecks, thereby increasing inference latency on resource-constrained edge devices. In contrast, the proposed parallel design ensures that high-resolution local textural cues extracted by the MobileNetV3 backbone are preserved and concurrently fused with the global spatial dependencies modeled by the Transformer branch. This architectural synergy enables the network to construct a discriminative, multi-scale feature representation without incurring the parameter inflation or operational overhead associated with conventional sequential stacking.

This structural fusion is further augmented by the CutMix regularization strategy, which guides the model to prioritize intrinsic pathological markers over general image contours. Consequently, the framework effectively mitigates the misclassification of visually analogous diseases. As demonstrated in the comparative confusion matrices (**Figure 6B**), the recognition accuracy for Brown Blight—a category frequently misidentified as Algal Leaf Spot by the monolithic CNN baseline—exhibited significant improvement. These findings substantiate that integrating holistic spatial context via a parallel fusion mechanism is critical for resolving localized visual ambiguities in complex phytopathological diagnostic tasks.

### 4.3 Computational parsimony and potential for edge deployment

The successful translation of deep learning diagnostic models from controlled laboratory environments to real-world agricultural applications is frequently hindered by substantial computational overhead. Continuous, real-time monitoring within modern smart tea plantations requires algorithms capable of executing rapid inference on mobile or embedded edge devices (Jeyabose et al., 2022; Sharath et al., 2024), which inherently possess limited computational resources and power budgets. The proposed GL-MobFormer achieves an optimal trade-off between diagnostic precision and operational efficiency.

As detailed in the comprehensive horizontal evaluation (**Figure 8**, **Table 4**), the model operates with a minimal computational footprint of merely 0.33 G FLOPs and requires only 10.53 M parameters. This represents a drastic reduction in hardware demands when compared to standard heavyweight architectures evaluated in this study, such as ResNet50 (4.13 G FLOPs) or VGG16 (15.52 G FLOPs). By strategically confining the computationally intensive self-attention operations to a lightweight encoder module, the proposed framework successfully circumvents the parametric bloat typical of pure ViT models. This computational efficiency ensures that the system can be seamlessly integrated into embedded agricultural diagnostic terminals without compromising high-fidelity disease identification.

### 4.4 Limitations and future perspectives

While the proposed GL-MobFormer framework exhibits robust overall performance, certain constraints inherent to the current study warrant further investigation. A primary diagnostic challenge remains the reliable differentiation of highly analogous pathological signatures during their incipient stages, such as the pre-necrotic visual cues shared by Brown Blight and Gray Blight. Since conventional RGB-based vision relies exclusively on manifested optical changes, early-stage latent infections may remain visually indistinguishable. Future iterations of this pipeline should explore the integration of multi-spectral imaging (Behmann et al., 2014). Such modalities could capture pre-symptomatic biochemical alterations—including localized chlorophyll degradation—long before necrotic lesions become visually apparent (Mahlein, 2016).

Furthermore, it must be acknowledged that the teaLeafBD dataset primarily features relatively uniform backgrounds, which may not fully encapsulate the stochastic complexities of “in-the-wild” tea plantations. Although the incorporation of the CutMix strategy was specifically intended to mitigate background dependency by guiding the model to focus on intrinsic pathological motifs (Yun et al., 2019), the transition to genuinely unstructured environments remains a critical hurdle. To ensure global applicability, subsequent research must prioritize multi-center field validation. Expanding the data corpus to encompass variable illumination, complex canopy occlusions, and diverse microclimates will be essential for refining the model’s structural robustness prior to large-scale agronomic deployment.

Regarding the dataset imbalance, it is noted that targeted remedies such as class-weighted loss or synthetic oversampling were not explicitly implemented in this study. However, the CutMix strategy partially mitigated this issue by synthesizing diverse training patterns, effectively enriching the feature space for minority classes through patch-level interpolation. Future iterations of the GL-MobFormer framework could further integrate Cost-Sensitive Learning or SMOTE-based augmentation to enhance sensitivity to rare pathological signatures in even more imbalanced real-world scenarios.

## 5 Conclusion

This study proposed GL-MobFormer, a hybrid deep learning framework engineered to balance high diagnostic sensitivity with computational efficiency for the precise identification of tea leaf phytopathogens in complex agricultural environments. To address the inherent limitations of standard Convolutional Neural Networks in capturing long-range global dependencies, alongside the high computational demands of pure Vision Transformers, this research developed an effective dual-branch architecture. Empirical evaluations demonstrate that integrating the local feature extraction of MobileNetV3 with the global contextual modeling of a Transformer Encoder, further augmented by the CutMix strategy, significantly enhances the network’s discriminative capacity and generalization robustness.

Evaluated on the hold-out test set, GL-MobFormer achieved a classification accuracy of 95.13% while maintaining a low operational footprint of merely 0.33 G FLOPs. When benchmarked against mainstream architectures such as ResNet50, VGG16, and pure ViT, the proposed model delivered optimal performance across all primary evaluation metrics (Accuracy, F1-score, MCC), effectively overcoming the traditional trade-off between lightweight design and diagnostic precision. Furthermore, this study bridges the gap between high-performance deep learning and model transparency. Beyond achieving high diagnostic accuracy, the integration of a dual-layer interpretability framework—validated by a 70.61% average confidence drop in quantitative occlusion tests—empirically confirms that GL-MobFormer accurately prioritizes biologically relevant lesion features. This rigorous validation ensures that the model provides not only accurate but also explainable results, satisfying the stringent requirements for accountability in precision agriculture. In conclusion, GL-MobFormer represents a robust, efficient, and trustworthy solution for real-time tea disease monitoring on resource-constrained edge devices.

In summary, GL-MobFormer presents an efficient and trustworthy solution for real-time phytosanitary monitoring. Future research will prioritize the validation of the model against unstructured field imagery characterized by complex lighting and occlusions, and explore class-balanced loss functions to further refine performance under natural population imbalances. Additionally, the integration of multi-spectral sensing will be investigated to extend the framework’s capability toward the detection of early-stage, asymptomatic infections in smart tea plantations.

## Data Availability

The original contributions presented in the study are included in the article/supplementary material. Further inquiries can be directed to the corresponding authors.
